# A Matter of Caution: Coagulation Parameters in COVID-19 Do Not Differ from Patients with Ruled-Out SARS-CoV-2 Infection in the Emergency Department

**DOI:** 10.1055/s-0040-1722612

**Published:** 2021-02-06

**Authors:** Wolfgang Bauer, Noa Galtung, Nick Neuwinger, Lutz Kaufner, Elisabeth Langer, Rajan Somasundaram, Rudolf Tauber, Kai Kappert

**Affiliations:** 1 Department of Emergency Medicine, Charité—Universitätsmedizin Berlin, Freie Universität Berlin, Humboldt-Universität zu Berlin, and Berlin Institute of Health, Berlin, Germany; 2 Institute of Laboratory Medicine, Clinical Chemistry and Pathobiochemistry, Charité—Universitätsmedizin Berlin, Freie Universität Berlin, Humboldt-Universität zu Berlin, and Berlin Institute of Health, Berlin, Germany; 3 Labor Berlin—Charité Vivantes GmbH, Berlin, Germany; 4 Department of Anesthesiology and Operative Intensive Care Medicine, Charité—Universitätsmedizin Berlin, Freie Universität Berlin, Humboldt—Universität zu Berlin, and Berlin Institute of Health, Berlin, Germany

**Keywords:** D-dimer, COVID-19, risk stratification, emergency department, intensive care unit

## Abstract

COVID-19 (coronavirus disease 2019) patients often show excessive activation of coagulation, associated with increased risk of thrombosis. However, the diagnostic value of coagulation at initial clinical evaluation is not clear. We present an in-depth analysis of coagulation in patients presenting to the emergency department (ED) with suspected COVID-19. *N* = 58 patients with clinically suspected COVID-19 in the ED were enrolled. *N* = 17 subsequently tested positive using SARS-CoV-2 (severe acute respiratory syndrome coronavirus 2) polymerase chain reaction (PCR) swabs, while in *n* = 41 COVID-19 was ruled-out. We analyzed both standard and extended coagulation parameters, including thromboplastin time (INR), activated partial thromboplastin time (aPTT), antithrombin, plasminogen, plasminogen activator inhibitor-1 (PAI-1), D-dimers, and fibrinogen at admission, as well as α2-antiplasmin, activated protein C -resistance, factor V, lupus anticoagulant, protein C, protein S, and von Willebrand diagnostics. These data, as well as mortality and further laboratory parameters, were compared across groups based on COVID-19 diagnosis and severity of disease. In patients with COVID-19, we detected frequent clotting abnormalities, including D-dimers. The comparison cohort in the ED, however, showed similarly altered coagulation. Furthermore, parameters previously shown to distinguish between severe and moderate COVID-19 courses, such as platelets, plasminogen, fibrinogen, aPTT, INR, and antithrombin, as well as multiple nonroutine coagulation analytes showed no significant differences between patients with and without COVID-19 when presenting to the ED. At admission to the ED the prevalence of coagulopathy in patients with COVID-19 is high, yet comparable to the non-COVID-19 cohort presenting with respiratory symptoms. Nevertheless, coagulopathy might worsen during disease progression with the need of subsequent risk stratification.

## Introduction

The 2019 coronavirus disease (COVID-19) caused by the severe acute respiratory syndrome coronavirus 2 (SARS-CoV-2) virus impacts on various aspects of patient care, including coagulation management. During disease progression, COVID-19 patients often display activation of coagulation and endothelial dysfunction associated with elevated risk of thrombotic events and fatal outcome. 1 2 3 4 5 A high proportion of patients who died (71.4%) showed signs of disseminated intravascular coagulation, but only 0.6% of surviving patients. 3 Bilaloglu et al reported an incidence of 16% thrombotic events among hospitalized patients. 6 Among patients with COVID-19 being hospitalized or admitted to intensive care units (ICU), high rates of thrombotic events, ranging from 31 to 58.6%, were documented. 7 8 In a Dutch study evaluating 184 critically ill patients with COVID-19 pneumonia, the cumulative incidence of thromboembolic complication over a median observation period of 7 days was 31% (27% venous and 4% arterial events) despite standard prophylaxis with low-molecular-weight heparin. 7

Patients with a severe course of disease showed significantly increased D-dimer concentrations already at admission. 3 The D-dimer concentration increased massively in severe courses 9 10 11 12 and was suggested as a predictor for the development of severe acute respiratory distress syndrome (ARDS). 13 Additionally, increased levels of fibrin degradation products as well as prolonged prothrombin time (PT) were found in nonsurvivors compared with survivors 3 or compared with healthy controls. 14 In contrast, data for antithrombin (AT) values are inconsistent. Both decreased AT and nonaffected AT levels were reported in several investigations. 3 14 The activated partial thromboplastin time (aPTT) at admission and before anticoagulation treatment might be slightly prolonged above the normal range in moderate, but not severe cases. 11 However, of 216 COVID-19 patients with ARDS, 44 (20%) showed aPTT prolongation without any changes of coagulation factors VIII, IX, XI, and XII, but with a significantly increased fraction of lupus anticoagulant positive patients compared with a historic healthy control. 15 In a retrospective analysis of 22 severe compared with 91 nonsevere cases, severe patients displayed a higher level of fibrinogen, as well as D-dimer at admission with increasing difference during disease progression. 16 Similar to severe infection disorders the fibrinogen to albumin ratio was increased, while platelet counts were decreased revealing both as independent risk factors for severity of COVID-19. 16

Of note, the majority of these studies have been limited to identifying differences in standard coagulation parameters between mild and severe courses of COVID-19 during hospital stay or in comparison to healthy individuals. Here, we present a comprehensive coagulation analysis in patients with clinically suspected COVID-19 at a defined and early time point, namely at the admission to the ED, to investigate the question whether COVID-19 patients show specific coagulation disorders.

## Methods

### Study Design and Study Population

**Fig. 1:**
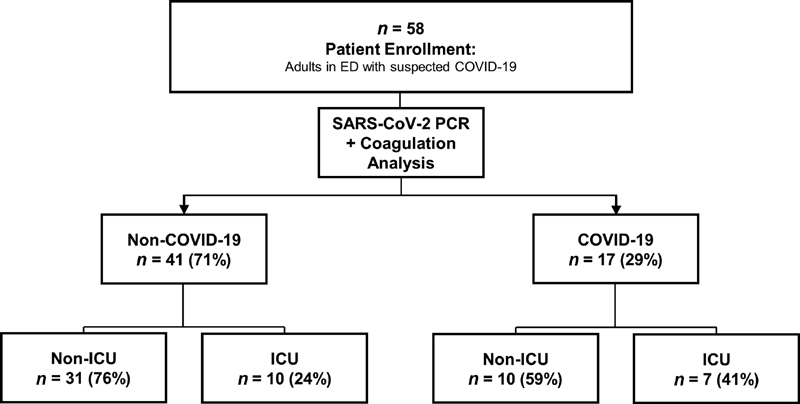
Flowchart of patient enrollment and division into four groups by COVID-19 status and ICU treatment for comparison. COVID-19, coronavirus disease 2019; ICU, intensive care unit.

We performed a noninterventional, observational study at the emergency department (ED) of the university hospital Charité—Universitätsmedizin Berlin, Campus Benjamin Franklin in Berlin, Germany. The study was approved by the institutional review board at Charité—Universitätsmedizin Berlin, Campus Benjamin Franklin in Berlin, Germany (no. EA4/167/18) and written informed consent was obtained from each study participant or patient's legal guardian before enrollment. From March 2020 until June 2020 we enrolled a consecutive sample of 58 adult patients (≥18 years) presenting to the ED with clinically suspected COVID-19. Patients were evaluated for COVID-19 by SARS-CoV-2 PCR or polymerase chain reaction in pharyngeal swabs. PCR results were obtained within 48 hour and did not impact on treatment at admission. Among the 58 patients, 17 were diagnosed with COVID-19, and 41 were tested negative for SARS-CoV-2. Of the 41 negatively tested patients, all underwent radiological imaging, of which 40 were chest CTs and one was a chest X-ray. Of the 41 patients who tested negative for SARS-CoV-2, 10 had inconclusive radiological findings, of which four were consequently tested for SARS-CoV-2 using sputum and/or bronchoalveolar lavage, all of which remained negative. The remaining six patients with negative PCR results and inconclusive radiological findings all received an alternative, plausible diagnosis explaining the radiology and were, as a result, released from isolation and quarantine. All enrolled patients were examined, diagnosed, and treated according to the standard of care, including laboratory blood cell count and clinical chemistry analyses. Blood was isolated for immediate routine analyses from all patients according to standard of care. From both groups we monitored consecutive disease progression, categorized by ICU admission versus non-ICU treatment. Clinical course of all patients is outlined in Fig. 1 . EDTA blood was drawn from all patients for blood counts. Serum and citrate plasma samples were isolated in serum and sodium citrate (3.2%) tubes (all tubes from Greiner, Bio-One, Kremsmünster, Austria), centrifuged within 30 minutes at 2,200 g for 10 minutes. Citrate plasma aliquots for extended coagulation analyses were subjected to a second centrifugation at 2,200 g for 10 minutes and stored immediately at −80°C until further use.

### Standard of Care Blood Analyses

Laboratory values were measured as standard of care at the time of admission to the ED: blood count—hemoglobin (Hb), white blood cells (WBC), red blood cells (RBC), mean corpuscular volume (MCV), mean corpuscular hemoglobin (MCH), mean corpuscular Hb concentration (MCHC), platelets (PLT), and mean platelet volume (MPV) on a Sysmex XN-10 hematology analyzer within an XN-2000 configuration in EDTA tubes (Greiner, Bio-One, Kremsmünster, Austria); clinical chemistry—aspartate aminotransferase (AST), alanine aminotransferase (ALT), gamma-glutamyl transferase (GGT), alkaline phosphatase (AP), lactate dehydrogenase (LDH), lipase, creatine kinase (CK), CK-MB, creatinine, indirect and direct bilirubin, C-reactive protein (CRP), myoglobin, blood urea (UREA), and procalcitonin (PCT) were determined applying Roche cobas c701, e602, and e801 analyzers within a cobas 6,000/8,000 configuration in heparin plasma tubes (Greiner, Bio-One, Kremsmünster, Austria). Sodium, potassium, calcium, chloride, glucose, and lactate were measured directly in the ED on an ABL800 FLEX (Radiometer, Copenhagen, Denmark) in venous blood in gas sampling syringes (PICO50, Arterial Blood Sampler 2 mL, Radiometer Medical ApS, Brønshøj, Denmark).

### Coagulation Analyses

The citrate plasma was used for coagulation testing. Measurements of lupus anticoagulant sensitive aPTT (coagulometry) and PT/international normalized ratio (PT/INR, coagulometry) were performed at presentation to the ED as standard of care. Furthermore, extending coagulation analyses were performed in isolated citrate plasma and included: plasminogen (photometry, chromogenic substrate), lupus anticoagulant insensitive PTT-FS (coagulometry), thrombin time (TT, coagulometry), fibrinogen (coagulometry according to Clauss), AT (photometry, chromogenic substrate), D-dimer (turbidimetry), α2 antiplasmin (photometry, chromogenic substrate), lupus anticoagulant (coagulometry, Dilute Russell viper venom time, normalized ratio), factor V (coagulometry), protein C activity (coagulometry), protein S activity (coagulometry), resistance against activated protein C (APCR, coagulometry), and vWF antigen (latex-enhanced immunoassay). All coagulation analyses were quantified using the STAR MAX analyzer (Stago Germany GmbH, Düsseldorf). The vWF Ristocetin cofactor activity (RICOF) was determined by test thrombocyte agglutination on the Behring Coagulation System XP (Siemens Healthineers, Erlangen, Germany). Tissue plasminogen activator (T-PA, immunological, ref. TC12007), and plasminogen activator inhibitor-I (PAI-1, immunological, ref. TC12075) were measured by antigen enzyme-linked immunosorbent assay (ELISA, both Technoclone, Vienna, Austria). All coagulation analyses were performed at our institution's laboratory (Labor Berlin—Charité Vivantes GmbH, Berlin, Germany) and are presented with standard reference range (as per our single-center laboratory). This also accounts for analytes, which are presented as percentages in comparison to calibration curves applying values obtained from the standardized and/or international references for laboratory testing within our hospital system for all patients.

### Statistics

Data on continuous variables are expressed as median and interquartile range, and *p* -values calculated using Mann-Whitney U test. Categorical data are expressed as frequency and column percentage and compared using Fisher's exact test. *p* < 0.05 was considered statistically significant for all analyses. Statistical analyses were done using R version 3.6.3.

## Results

### Baseline Data

Table 1 shows the patients' general characteristics and clinical outcome (30 and 90 d mortality). We included a total of 58 adult consecutively enrolled patients who presented in the ED with clinical signs of COVID-19. Within 48 hours patients were tested for SARS-CoV-2 using PCR in pharyngeal swabs. Seventeen patients were diagnosed with COVID-19, while 41 patients were ruled-out. Medical history including symptoms at presentation was taken from all patients. Blood was evaluated for blood counts and clinical chemistry. No differences were evident for age, sex, body mass index (BMI), kidney disease, coronary heart disease, hypertension, type 2 diabetes, chronic obstructive pulmonary disease, bronchial asthma, nicotine use as well as malignant disease at presentation. In addition, no differences were evident with regard to current oral anticoagulation ( Table 1 ). The median age of the 17 COVID-19 patients was 70.1 years (58.3 males and 70.5 females), and the median age of the non-COVID-19 patients was 69.8 years (71.0 males and 68.5 females). There were no significant differences between the two groups in regards to sex and age ( *p* = 0.15, and *p* = 0.44, respectively) as well as BMI ( *p* = 0.29). According to patient cohorts, as grouped in Fig. 1 , the only significant differences were evident with regard to the prevalence of tachypnea in COVID-19 ICU patients (57%) compared with non-ICU COVID-19 patients (0%), since tachypnea is essentially present in imminent respiratory failure. Finally, 30- and 90-day mortality showed no significant difference between COVID-19 and non-COVID-19 patients.

**Table 1:** Demographics

	All	Non-COVID-19			COVID-19			*p* -Values		
	( *n* = 58)	All ( *n* = 41)	Non-ICU ( *n* = 31)	ICU ( *n* = 10)	All ( *n* = 17)	Non-ICU ( *n* = 10)	ICU ( *n* = 7)	COVID-19 vs. non-COVID-19	ICU-COVID-19 vs. non-ICU-COVID-19	ICU-COVID-19 vs. ICU-non-COVID-19
Age (years)	70.0 (54.7, 76.4)	69.8 (54.5, 79.1)	68.8 (50.2, 77.5)	75.6 (66.2, 87.2)	70.1 (55.6, 72.0)	63.7 (52.5, 71.0)	71.9 (57.5, 76.8)	0.44	0.27	0.27
Female	28 (48%)	17 (41%)	14 (45%)	3 (30%)	11 (65%)	6 (60%)	5 (71%)	0.15	1.00	0.15
BMI (kg/m ^2^ )	24.5 (22.13, 30.2)	24.1 (22.1, 28.5)	24.2 (21.93, 28.0)	24.1 (23.6, 28.9)	27.0 (22.3, 30.5)	24.9 (21.9, 29.6)	29.4 (26.4, 30.9)	0.29	0.19	0.19
*Comorbidity*										
Chronic kidney disease	8 (14%)	7 (17%)	5 (16%)	2 (20%)	1 (6%)	1 (10%)	0 (0%)	0.42	1.00	0.49
Coronary heart disease	12 (21%)	11 (27%)	6 (19%)	5 (50%)	1 (6%)	0 (0%)	1 (14%)	0.09	0.41	0.30
Hypertension	34 (60%)	25 (62%)	19 (63%)	6 (60%)	9 (53%)	5 (50%)	4 (57%)	0.56	1.00	1.00
Type 2 diabetes	13 (22%)	8 (20%)	5 (16%)	3 (30%)	5 (29%)	4 (40%)	1 (14%)	0.49	0.34	0.60
COPD	7 (12%)	6 (15%)	5 (16%)	1 (10%)	1 (6%)	0 (0%)	1 (14%)	0.66	0.41	1.00
Asthma	5 (9%)	4 (10%)	3 (10%)	1 (10%)	1 (6%)	0 (0%)	1 (14%)	1.00	0.41	1.00
Current smoker	9 (16%)	9 (22%)	8 (26%)	1 (10%)	0 (0%)	0 (0%)	0 (0%)	0.10	0.64	0.76
Malignant disease	12 (21%)	9 (22%)	6 (19%)	3 (30%)	3 (18%)	2 (20%)	1 (14%)	1.00	1.00	0.60
Oral anticoagulation	7 (12%)	5 (12%)	3 (10%)	2 (20%)	2 (12%)	0 (0%)	2 (29%)	0.17	0.15	0.56
*Presentation*										
Cough	20 (34%)	15 (37%)	12 (39%)	3 (30%)	5 (29%)	4 (40%)	1 (14%)	0.76	0.34	0.60
Fever	39 (67%)	25 (61%)	19 (61%)	6 (60%)	14 (82%)	9 (90%)	5 (71%)	0.14	0.54	1.00
Flu-like symptoms	34 (59%)	21 (51%)	16 (52%)	5 (50%)	13 (76%)	8 (80%)	5 (71%)	0.09	1.00	0.62
Hypotension	5 (9%)	3 (7%)	3 (10%)	0 (0%)	2 (12%)	0 (0%)	2 (29%)	0.62	0.15	0.15
Tachypnea	15 (26%)	11 (27%)	8 (26%)	3 (30%)	4 (24%)	0 (0%)	4 (57%)	1.00	**0.01**	0.35
Altered mentation	3 (5%)	2 (5%)	2 (6%)	0 (0%)	1 (6%)	0 (0%)	1 (14%)	1.00	0.41	0.41
*Outcome*										
30-day mortality	2 (3%)	1 (2%)	0 (0%)	1 (10%)	1 (6%)	0 (0%)	1 (14%)	0.50	0.41	1.00
90-day mortality	4 (7%)	2 (5%)	1 (3%)	1 (10%)	2 (12%)	0 (0%)	2 (29%)	0.57	0.15	0.54

Abbreviations: BMI, body mass index; COPD, chronic obstructive pulmonary disease; COVID, coronavirus disease 2019; ICU, intensive care unit.

Note: Continuous variables are represented with median and IQR, nominal variables with count and column percentage; *p* -values are calculated with Mann-Whitney U test for continuous variables and Fisher's Exact test for nominal variables.

### Clinical Laboratory Data

We performed initial analyses of hematological and clinical chemistry parameters in our cohort, as shown in Table 2 . Analytes of the complete blood counts were not significantly different between the two groups with regard RBC, PLT, Hb, as well as index parameters including MCV, MCH, MCHC, and MPV. However, in line with recent findings, WBC was significantly lower in the COVID-19 cohort ( *p* < 0.01), while the subgroups of ICU versus non-ICU COVID patients did not show significant differences.

**Table 2:** Clinical chemistry and hematology

	All	Non-COVID-19			COVID-19			*p* -Values		
	( *n* = 58)	All ( *n* = 41)	Non-ICU ( *n* = 31)	ICU ( *n* = 10)	All ( *n* = 17)	Non-ICU ( *n* = 10)	ICU ( *n* = 7)	COVID-19 vs. non-COVID-19	ICU-COVID-19 vs. non-ICU-COVID-19	ICU-COVID-19 vs. ICU-non-COVID-19
CRP (mg/L)	46.5 (12.7, 105.1)	56.8 (12.2, 105.2)	55.4 (10.1, 105.0)	65.4 (16.4, 100.3)	36.6 (18.7, 77.9)	36.0 (21.1, 56.7)	77.9 (19.2, 206.7)	1.00	0.42	0.67
CRP >REF	50 (86%)	35 (85%)	26 (84%)	9 (90%)	15 (88%)	9 (90%)	6 (86%)	1.00	1.00	1.00
PCT (µg/L)	57; 0.1 (0.1, 0.3)	40; 0.1 (0.1, 0.4)	0.1 (0.0, 0.3)	9; 0.1 (0.1, 0.7)	0.1 (0.1, 0.2)	0.1 (0.1, 0.1)	0.1 (0.1, 0.5)	0.39	0.77	0.43
PCT >REF	10 (18%)	8 (20%)	5 (16%)	3 (33%)	2 (12%)	0 (0%)	2 (29%)	0.71	0.15	1.00
eGFR	68.0 (49.0, 90.0)	67.0 (50.0, 90.0)	77.0 (56.5, 90.0)	51.5 (25.8, 60.2)	74.0 (42.0, 90.0)	75.0 (48.8, 89.8)	60.0 (42.0, 83.0)	0.95	0.92	0.31
UREA (mg/dL)	47; 33.0 (22.5, 64.0)	32; 38.5 (23.8, 64.0)	25; 27.0 (23.0, 50.0)	7; 64.0 (52.0, 92.5)	15; 29.0 (18.5, 52.0)	8; 29.0 (20.8, 35.8)	29.0 (17.5, 69.5)	0.25	0.91	0.11
LDH (U/L)	51; 301.0 (244.5, 410.5)	35; 284.0 (241.0, 404.0)	27; 261.0 (241.0, 367.0)	8; 385.5 (318.0, 445.8)	16; 337.5 (258.8, 475.2)	317.0 (231.0, 342.0)	6; 509.0 (312.5, 986.0)	0.39	0.09	0.28
LDH >REF	35 (69%)	22 (63%)	16 (59%)	6 (75%)	13 (81%)	7 (70%)	6 (100%)	0.33	0.25	0.47
CK (U/L)	53; 104.0 (56.0, 149.0)	38; 86.5 (55.2, 147.8)	30; 72.0 (52.5, 134.2)	8; 134.5 (83.0, 188.2)	15; 120.0 (96.0, 148.5)	8; 101.5 (79.5, 213.0)	129.0 (111.0, 133.5)	0.34	0.52	0.77
CK >REF	9 (17%)	6 (16%)	4 (13%)	2 (25%)	3 (20%)	2 (25%)	1 (14%)	0.70	1.00	1.00
AST (U/L)	51; 34.0 (27.5, 54.5)	35; 31.0 (25.0, 54.5)	27; 31.0 (25.0, 51.5)	8; 32.0 (29.2, 71.2)	16; 44.0 (33.2, 55.5)	9; 40.0 (34.0, 49.0)	49.0 (37.5, 74.5)	0.12	0.34	0.45
AST >REF	19 (37%)	11 (31%)	8 (30%)	3 (38%)	8 (50%)	3 (33%)	5 (71%)	0.23	0.31	0.31
ALT (U/L)	52; 24.0 (18.8, 37.2)	36; 22.0 (17.0, 36.2)	28; 23.5 (16.5, 38.8)	8; 20.5 (18.5, 23.0)	16; 31.5 (20.0, 38.8)	9; 30.0 (19.0, 36.0)	36.0 (31.0, 61.0)	0.10	0.18	**0.04**
ALT >REF	14 (27%)	8 (22%)	7 (25%)	1 (12%)	6 (38%)	2 (22%)	4 (57%)	0.32	0.30	0.12
GGT (U/L)	50; 46.0 (27.5, 108.0)	34; 42.0 (24.5, 92.2)	26; 42.0 (24.0, 108.0)	8; 38.5 (30.0, 47.5)	16; 61.0 (36.0, 156.2)	9; 71.0 (33.0, 243.0)	57.0 (42.5, 102.5)	0.10	0.76	0.20
GGT >REF	24 (48%)	14 (41%)	12 (46%)	2 (25%)	10 (62%)	5 (56%)	5 (71%)	0.23	0.63	0.13
AP (U/L)	48; 81.5 (62.5, 109.2)	32; 87.0 (70.0, 118.2)	25; 88.0 (67.0, 118.0)	7; 85.0 (81.5, 105.5)	16; 62.5 (49.5, 85.0)	9; 60.0 (48.0, 88.0)	66.0 (61.5, 78.5)	**<0.01**	0.46	0.13
AP >REF	11 (23%)	9 (28%)	8 (32%)	1 (14%)	2 (12%)	2 (22%)	0 (0%)	0.29	0.48	1.00
LIP (U/L)	48; 34.0 (21.5, 48.8)	32; 29.0 (19.0, 41.0)	25; 27.0 (18.0, 36.0)	7; 33.0 (25.0, 41.5)	16; 48.5 (36.8, 69.5)	9; 53.0 (43.0, 69.0)	40.0 (34.5, 65.0)	**<0.01**	0.76	0.37
LIP >REF	8 (17%)	2 (6%)	2 (8%)	0 (0%)	6 (38%)	4 (44%)	2 (29%)	**0.01**	0.63	0.46
TBIL (mg/dL)	55; 0.6 (0.4, 0.8)	39; 0.6 (0.4, 0.9)	30; 0.6 (0.4, 0.9)	9; 1.0 (0.5, 1.1)	16; 0.4 (0.3, 0.6)	9; 0.4 (0.3, 0.5)	0.4 (0.3, 0.7)	**0.03**	0.61	0.19
TBIL >REF	5 (9%)	5 (13%)	4 (13%)	1 (11%)	0 (0%)	0 (0%)	0 (0%)	0.31	1.00	1.00
TSHB (mU/L)	56; 1.2 (0.7, 2.0)	39; 1.5 (0.7, 2.0)	29; 1.5 (0.7, 2.0)	1.4 (0.9, 2.1)	1.1 (0.6, 1.4)	1.2 (0.8, 1.5)	1.0 (0.4, 1.3)	0.27	0.56	0.23
Na (mmol/L)	137.0 (133.2, 139.0)	138.0 (135.0, 139.0)	137.0 (134.5, 139.5)	138.0 (136.2, 139.0)	135.0 (133.0, 139.0)	135.0 (133.2, 137.5)	137.0 (132.5, 139.0)	0.19	1.00	0.69
K (mmol/L)	3.9 (3.7, 4.2)	4.0 (3.7, 4.3)	3.9 (3.7, 4.3)	4.2 (4.1, 4.3)	3.9 (3.7, 4.0)	4.0 (3.8, 4.1)	3.8 (3.4, 3.8)	0.16	0.20	**0.02**
Ca (mmol/L)	57; 1.2 (1.1, 1.2)	40; 1.2 (1.1, 1.2)	30; 1.2 (1.2, 1.2)	1.2 (1.1, 1.2)	1.1 (1.1, 1.2)	1.1 (1.1, 1.2)	1.1 (1.1, 1.3)	0.64	0.73	0.62
Cl (mmol/L)	50; 104.0 (101.0, 107.0)	35; 104.0 (101.0, 107.0)	25; 104.0 (102.0, 108.0)	103.5 (100.2, 104.8)	15; 103.0 (101.0, 106.5)	8; 102.5 (101.0, 104.0)	105.0 (100.5, 108.0)	0.58	0.91	0.43
Glucose (mg/dL)	119.0 (104.2, 144.8)	118.0 (104.0, 127.0)	115.0 (104.5, 126.5)	122.5 (108.0, 135.2)	120.0 (107.0, 186.0)	116.5 (102.5, 163.8)	186.0 (110.0, 239.5)	0.28	0.27	0.36
Lactate (mg/dL)	14.2 (11.0, 18.7)	13.0 (11.0, 18.0)	13.0 (11.0, 18.0)	14.0 (11.2, 20.6)	15.0 (12.0, 21.0)	12.5 (11.1, 14.6)	22.0 (19.5, 32.5)	0.47	**<0.01**	0.06
Lactate >REF	12 (21%)	7 (17%)	4 (13%)	3 (30%)	5 (29%)	0 (0%)	5 (71%)	0.31	**<0.01**	0.15
*Hematology*										
WBC (/nL)	57; 8.1 (6.0, 12.2)	9.9 (7.1, 12.4)	9.5 (6.7, 12.3)	10.1 (8.1, 14.0)	16; 5.7 (4.6, 7.3)	9; 5.5 (4.9, 5.9)	9.4 (4.9, 11.3)	**<0.01**	0.11	0.42
RBC (/pL)	57; 4.3 (3.7, 4.7)	4.3 (3.7, 4.7)	4.3 (3.8, 4.7)	4.2 (3.7, 4.4)	16; 4.4 (4.1, 4.9)	9; 4.8 (4.1, 4.9)	4.3 (4.2, 4.8)	0.19	0.87	0.24
Hb (g/dL)	57; 12.9 (11.3, 14.0)	12.8 (11.3, 14.0)	12.8 (11.3, 14.0)	12.8 (11.2, 13.8)	16; 13.2 (12.6, 13.9)	9; 13.1 (11.8, 14.2)	13.3 (13.0, 13.6)	0.32	0.79	0.41
MCV (fL)	57; 87.0 (84.0, 90.0)	87.0 (85.0, 90.0)	87.0 (84.5, 88.5)	91.0 (88.2, 92.8)	16; 85.5 (83.8, 89.2)	9; 84.0 (84.0, 86.0)	89.0 (85.5, 92.0)	0.34	0.29	0.62
MCH (pg)	57; 30.4 (28.9, 30.9)	30.4 (29.3, 31.0)	29.9 (29.2, 30.9)	30.5 (29.8, 31.8)	16; 29.8 (28.6, 30.9)	9; 28.9 (28.5, 30.8)	30.7 (29.6, 30.9)	0.66	0.52	1.00
MCHC (g/dL)	57; 34.5 (33.6, 35.2)	34.5 (33.6, 35.2)	34.6 (33.8, 35.3)	33.6 (32.6, 34.8)	16; 34.5 (33.8, 34.9)	9; 34.5 (34.0, 35.6)	34.5 (33.6, 34.5)	0.96	0.40	0.66
RDW (%)	57; 13.4 (12.7, 14.9)	13.5 (12.7, 14.9)	13.3 (12.7, 14.9)	14.6 (12.8, 15.5)	16; 13.4 (12.8, 14.1)	9; 13.3 (12.9, 13.5)	13.6 (12.7, 14.2)	0.36	0.79	0.46
PLT (/nL)	57; 226.0 (177.0, 282.0)	236.0 (180.0, 294.0)	241.0 (192.0, 298.0)	218.0 (153.8, 270.5)	16; 199.5 (157.5, 234.2)	9; 187.0 (161.0, 233.0)	208.0 (167.0, 233.0)	0.16	1.00	0.81
MPV (fL)	55; 10.1 (9.5, 10.6)	39; 9.8 (9.5, 10.8)	30; 9.7 (9.4, 10.6)	9; 10.1 (9.6, 11.3)	16; 10.3 (10.0, 10.6)	9; 10.2 (9.9, 10.6)	10.5 (10.1, 10.6)	0.20	0.49	0.96

Abbreviations: AP, alkaline phosphatase; ALT, alanine aminotransferase; AST, aspartate aminotransferase; CK, creatine kinase; COVID-19, coronavirus disease 2019; CRP, C-reactive protein; GGT, gamma-glutamyl transferase; Hb, hemoglobin; ICU, intensive care unit; LIP, lipase; MCH, mean corpuscular hemoglobin; MCHC, mean corpuscular hemoglobin concentration; MCV, mean corpuscular volume; MPV, mean platelet volume; PLT, platelets; RBC, red blood cells; TBIL, total bilirubin; TSHB, thyroid stimulating hormone in blood; WBC, white blood cells.

Note: Continuous variables are represented with median and IQR, nominal variables with frequency and column percentage (of valid cases). Variables denoted by “ > REF” present the frequency and percentage of cases with values above the references range. No variables in this table had cases with values below the lower limit, and as such, the frequencies and percentages of cases below the reference range are not shown. All abbreviations are listed in Supplementary Table S1 . Reference ranges are listed in the Supplementary Table S2 ; For continuous variables where not all cases have data, the number of valid cases is shown; *p-* values are calculated with Mann-Whitney *U* -test for continuous variables and Fisher's Exact test for nominal variables.

Concerning clinical chemistry data, measures of CRP, PCT, estimated glomerular filtration rate, UREA, LDH, CK, AST, ALT, GGT, thyroid stimulating hormone, Na, K, Ca, Cl, and glucose, were comparable between the two groups. However, we observed significantly higher values of lipase, both absolute and percentage of pathological values, in the COVID-19 group ( *p* ≤ 0.01, Table 2 ). AP also showed a statistically significant difference between COVID-19 and non-COVID-19 patients, however, these changes were largely below the clinically used cut-off. Total bilirubin also showed a similar tendency. In accordance with ICU admission criteria, we observed higher lactate levels at presentation among COVID-19 patients who were later admitted to ICU, than COVID-19 patients who were not.

### Coagulation Analyses

We next subjected citrate plasma from both patient subgroups to in-depth coagulation analyses ( Table 3 ). Surprisingly, values of D-dimer, which were shown to predict outcomes of hospitalized COVID-19 patients earlier, 9 11 17 were not different between all the subgroups, at patients' presentation to the ED, highlighting both the high frequency of coagulation alterations at admission and indicating the limited information of D-dimer values in the early stages of COVID-19 disease development. In detail, 73 versus 82% in non-COVID-19 and COVID-19 patients, respectively, which presented to the ED, displayed D-dimers above the cut-off.

**Table 3:** Coagulation

	All	Non-COVID-19			COVID-19			***p*** -Values		
	( *n* = 58)	All ( *n* = 41)	Non-ICU ( *n* = 31)	ICU ( *n* = 10)	All ( *n* = 17)	Non-ICU ( *n* = 10)	ICU ( *n* = 7)	COVID-19 vs. Non-COVID-19	ICU COVID-19 vs. Non-ICU-COVID	ICU-COVID vs. ICU-Non-COVID
DDIM (mg/L)	1.1 (0.5, 1.7)	1.1 (0.5, 1.7)	1.1 (0.5, 1.9)	1.0 (0.6, 1.4)	1.0 (0.6, 1.7)	1.1 (0.6, 1.6)	0.9 (0.8, 11.0)	0.84	0.96	0.73
DDIM >REF	44 (76%)	30 (73%)	22 (71%)	8 (80%)	14 (82%)	8 (80%)	6 (86%)	0.52	1.00	1.00
INR	56; 1.1 (1.0, 1.2)	40; 1.1 (1.1, 1.3)	30; 1.1 (1.1, 1.2)	1.2 (1.1, 1.7)	16; 1.1 (1.0, 1.1)	1.1 (1.0, 1.1)	6; 1.2 (1.0, 1.6)	0.08	0.23	0.55
INR >REF	13 (23%)	10 (25%)	6 (20%)	4 (40%)	3 (19%)	0 (0%)	3 (50%)	0.74	**0.04**	1.00
INR <REF	0 (0%)	0 (0%)	0 (0%)	0 (0%)	0 (0%)	0 (0%)	0 (0%)	–	–	–
aPTT (s)	34.6 (31.3, 40.8)	34.5 (31.2, 41.4)	34.5 (30.6, 39.1)	37.8 (33.9, 42.8)	34.7 (32.5, 37.1)	33.2 (31.6, 34.6)	36.8 (35.1, 116.5)	0.97	**0.04**	0.42
aPTT >REF	15 (26%)	12 (29%)	7 (23%)	5 (50%)	3 (18%)	0 (0%)	3 (43%)	0.51	0.051	1.00
aPTT <REF	0 (0%)	0 (0%)	0 (0%)	0 (0%)	0 (0%)	0 (0%)	0 (0%)	–	–	–
PTT-FS (s)	27.1 (24.7, 30.7)	27.4 (24.3, 32.0)	27.2 (24.0, 30.4)	30.6 (25.8, 39.2)	26.2 (25.3, 28.5)	26.3 (25.4, 27.3)	26.2 (24.3, 30.0)	0.74	0.92	0.33
TT (s)	10.3 (9.8, 10.8)	10.3 (9.6, 10.7)	10.2 (9.6, 10.7)	10.4 (9.9, 10.7)	10.6 (10.1, 10.9)	10.4 (10.0, 10.8)	10.7 (10.4, 11.6)	0.15	0.41	0.30
AT (%)	98.0 (92.0, 105.8)	96.0 (92.0, 105.0)	97.0 (94.0, 106.5)	94.0 (81.5, 102.8)	100.0 (92.0, 106.0)	103.5 (99.5, 109.8)	92.0 (89.5, 98.0)	0.49	0.07	0.92
AT >REF	2 (3%)	2 (5%)	2 (6%)	0 (0%)	0 (0%)	0 (0%)	0 (0%)	1.00	1.00	1.00
AT <REF	8 (14%)	7 (17%)	4 (13%)	3 (30%)	1 (6%)	0 (0%)	1 (14%)	0.42	0.41	0.60
F5 (%)	104.0 (86.5, 117.0)	100.0 (84.0, 108.0)	99.0 (83.5, 107.0)	101.5 (86.5, 117.5)	117.0 (105.0, 132.0)	114.5 (105.5, 130.8)	117.0 (96.0, 127.5)	**0.01**	0.92	0.54
F5 >REF	4 (7%)	3 (7%)	2 (6%)	1 (10%)	1 (6%)	0 (0%)	1 (14%)	1.00	0.41	1.00
F5 <REF	3 (5%)	3 (7%)	2 (6%)	1 (10%)	0 (0%)	0 (0%)	0 (0%)	0.55	1.00	1.00
FIB (g/L)	5.1 (3.9, 6.3)	5.0 (3.9, 6.5)	5.0 (3.9, 6.7)	5.1 (4.5, 6.2)	5.2 (3.6, 5.9)	5.2 (4.3, 5.8)	5.7 (2.9, 6.3)	0.82	0.89	0.89
FIB >REF	41 (71%)	29 (71%)	21 (68%)	8 (80%)	12 (71%)	8 (80%)	4 (57%)	1.00	0.59	0.25
PLG (%)	110.0 (92.2, 122.8)	109.0 (86.0, 118.0)	107.0 (89.0, 119.0)	110.0 (84.8, 117.0)	116.0 (98.0, 130.0)	126.5 (110.0, 133.0)	112.0 (82.5, 119.0)	0.16	0.11	0.81
PLG >REF	16 (28%)	8 (20%)	7 (23%)	1 (10%)	8 (47%)	6 (60%)	2 (29%)	0.052	0.33	0.54
PLG <REF	10 (17%)	7 (17%)	5 (16%)	2 (20%)	3 (18%)	1 (10%)	2 (29%)	1.00	0.54	1.00
A2AP (%)	98.0 (89.2, 106.8)	95.0 (87.0, 103.0)	98.0 (86.5, 103.0)	92.0 (90.0, 97.2)	106.0 (93.0, 112.0)	107.5 (101.8, 111.5)	93.0 (88.5, 113.5)	**0.04**	0.54	0.84
A2AP >REF	2 (3%)	1 (2%)	0 (0%)	1 (10%)	1 (6%)	0 (0%)	1 (14%)	0.50	0.41	1.00
A2A *p* < REF	6 (10%)	5 (12%)	4 (13%)	1 (10%)	1 (6%)	0 (0%)	1 (14%)	0.66	0.41	1.00
PROTC (%)	96.0 (70.2, 118.5)	92.0 (65.0, 119.0)	104.0 (64.5, 125.5)	77.5 (70.2, 91.5)	100.0 (77.0, 117.0)	112.5 (88.0, 120.8)	84.0 (77.0, 93.5)	0.53	0.24	0.59
PROTC >REF	7 (12%)	5 (12%)	5 (16%)	0 (0%)	2 (12%)	1 (10%)	1 (14%)	1.00	1.00	0.41
PROTC <REF	13 (22%)	11 (27%)	9 (29%)	2 (20%)	2 (12%)	1 (10%)	1 (14%)	0.31	1.00	1.00
PROTS (%)	86.5 (65.0, 110.2)	93.0 (72.0, 124.0)	93.0 (74.0, 127.5)	89.0 (62.0, 105.5)	74.0 (58.0, 87.0)	82.5 (63.2, 95.2)	73.0 (48.5, 79.5)	0.06	0.42	0.43
PROTS >REF	4 (7%)	3 (7%)	3 (10%)	0 (0%)	1 (6%)	0 (0%)	1 (14%)	1.00	0.41	0.41
PROTS <REF	15 (26%)	9 (22%)	7 (23%)	2 (20%)	6 (35%)	3 (30%)	3 (43%)	0.33	0.64	0.59
APCR >REF	3 (5%)	1 (2%)	1 (3%)	0 (0%)	2 (12%)	2 (20%)	0 (0%)	0.19	0.50	1.00
vWF-Ag (%)	243.5 (192.0, 330.5)	243.0 (188.0, 329.0)	276.0 (187.0, 348.0)	221.5 (192.5, 284.5)	244.0 (208.0, 346.0)	209.0 (195.2, 280.0)	256.0 (244.5, 382.5)	0.54	**0.04**	0.11
vWF-Ag >REF	51 (88%)	34 (83%)	24 (77%)	10 (100%)	17 (100%)	10 (100%)	7 (100%)	0.09	1.00	1.00
vWF-Ag <REF	0 (0%)	0 (0%)	0 (0%)	0 (0%)	0 (0%)	0 (0%)	0 (0%)	–	–	–
RICOF (%)	195.0 (155.5, 254.2)	206.0 (148.0, 272.0)	220.0 (147.0, 277.5)	186.5 (153.5, 233.0)	182.0 (167.0, 234.0)	177.0 (161.5, 198.2)	199.0 (171.5, 248.0)	0.59	0.46	0.42
RICOF >REF	41 (71%)	28 (68%)	22 (71%)	6 (60%)	13 (76%)	7 (70%)	6 (86%)	0.75	0.60	0.34
RICOF <REF	0 (0%)	0 (0%)	0 (0%)	0 (0%)	0 (0%)	0 (0%)	0 (0%)	–	–	–
T-PA (µg/L)	2.2 (1.4, 3.0)	1.8 (1.4, 2.5)	1.8 (1.3, 2.6)	2.4 (1.7, 2.5)	2.2 (1.7, 4.5)	1.8 (1.4, 2.2)	8.1 (2.5, 9.8)	0.34	**0.02**	0.06
T-PA >REF	6 (10%)	2 (5%)	2 (6%)	0 (0%)	4 (24%)	0 (0%)	4 (57%)	0.055	**0.01**	**0.01**
T-PA <REF	28 (48%)	21 (51%)	17 (55%)	4 (40%)	7 (41%)	6 (60%)	1 (14%)	0.57	0.13	0.34
PAI-1 (ng/mL)	15.0 (9.0, 33.8)	14.0 (8.0, 40.0)	12.0 (7.0, 22.5)	32.5 (12.0, 46.8)	20.0 (9.0, 28.0)	11.0 (9.0, 21.5)	28.0 (18.5, 53.5)	0.48	0.09	0.88
PAI-1 >REF	12 (21%)	9 (22%)	6 (19%)	3 (30%)	3 (18%)	1 (10%)	2 (29%)	1.00	0.54	1.00
PAI-1 <REF	8 (14%)	7 (17%)	7 (23%)	0 (0%)	1 (6%)	1 (10%)	0 (0%)	0.42	1.00	1.00
Lupus (% pos.)	7/47 (15%)	4/33 (12%)	3/27 (11%)	1/6 (17%)	3/14 (21%)	2 (20%)	1/4 (25%)	0.41	1.00	1.00

Abbreviations: APCR, resistance against activated protein-C; aPTT, activated partial thromboplastin time; COVID-19, coronavirus disease 2019; DDIM, D-dimers; FIB, fibrinogen; ICU, intensive care unit; PA, plasminogen activator; PAI, plasminogen activator inhibitor; PLG, plasminogen; PROTC, protein C; PROTS, protein S; PTT, partial thromboplastin time; RICOF, Ristocetin cofactor; TT, thromboplastin time; vWF, von Willebrand factor.

Note: Continuous variables are represented with median and IQR, nominal variables with frequency and column percentage (of valid cases). Variables denoted by “ > REF” or “ < REF” present the frequency and percentage of cases with values above or below the references range. All abbreviations are listed in Supplementary Table S1 . Reference ranges are shown in the Supplementary Table S2 ; For continuous variables where not all cases have data, the number of valid cases is shown; *p* -values are calculated with Mann-Whitney U test for continuous variables and Fisher's Exact test for nominal variables.

Further, plasminogen, INR, aPTT, fibrinogen, and AT did not differ between all subgroups. However, factor V displayed a statistically significant difference between COVID-19 and non-COVID-19 patients, although these changes were within the reference range.

Finally, we explored coagulation parameters that are not frequently analyzed within routine analyses or as standard-of-care at admission. To this end, also PAI-1, protein C activity and APCR, protein S activity, vWF antigen, and Ristocetin co-factor showed comparable values in all groups, both when analyzed as continuous variables and as categorical values (pathological/normal). Only for α-2 antiplasmin significantly higher values were detected in COVID-19 versus non-COVID-19 patients. T-PA showed high values among COVID-19 ICU patients ( *p* = 0.02 vs. non-ICU COVID-19 patients) which was also reflected in the percentage of cases above the cut-off (COVID-19, ICU = 57%, COVID-19, non-ICU = 0%, *p* = 0.01) also when compared with non-COVID-19, ICU patients (= 0%, *p* = 0.01).

Based on recent findings showing a high percentage (91%) of positivity for lupus anticoagulant in COVID-19 patients with prolonged aPTT, 15 we evaluated all patients for lupus anticoagulant. In total, we observed *n* = 7 (15% of the 47 cases with analyzable samples) with positive lupus anticoagulant, but no significantly higher frequencies in the COVID-19 group compared with the non-COVID-19 group ( *p* = 0.41).

**Fig. 2:**
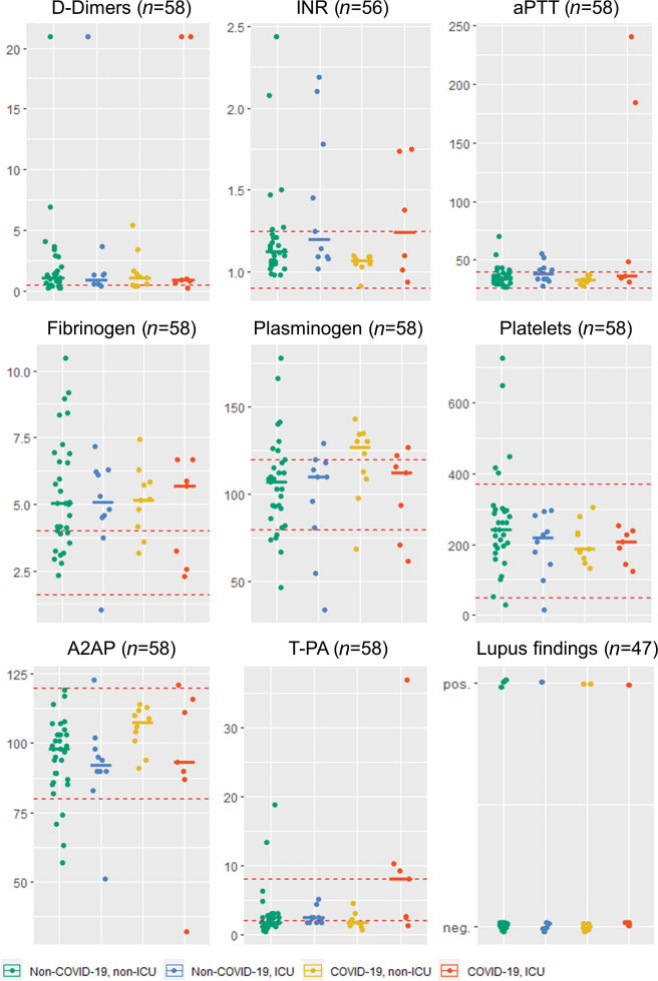
Jitter plot of coagulatory parameters by COVID-19 status and ICU treatment, as grouped in Fig. 1 . Red dashed lines represent cut-offs for reference ranges, as detailed in Supplementary Table S2 . Solid bars represent the median value for each group. COVID-19, coronavirus disease 2019; ICU, intensive care unit.

Taken together, at admission COVID-19 patients predominantly present a coagulation status that largely corresponds to a patient cohort in the ED with comparable symptoms but without detection of SARS-CoV-2 ( Fig. 2 ).

## Discussion

Most of the studies that addressed coagulation disorders in COVID-19 patients focused on risk stratification between mild and severe courses of the disease. In our study we investigated whether, when presented at the ED with a clinical suspicion of COVID-19, patients with later confirmed SARS-CoV-2 infection show different coagulation disorders as compared with patients later tested negative. The question was studied in a group of patients who presented to the ED with signs of infection such as respiratory distress, fever, and malaise. Using SARS-CoV-2 PCR, COVID-19 was confirmed in *n* = 17 patients but was ruled out in *n* = 41. The enrollment process led to four study groups ( Fig. 1 ), i.e., COVID-19 and non-COVID-19 patients each with or without subsequent ICU treatment. All four groups were very similar with respect to demographics and comorbidities. A large proportion (91%) of all enrolled patients was subsequently hospitalized. At presentation to the ED, we observed only very subtle differences in blood counts, in clinical chemistry parameters, and especially coagulation across all four groups. These findings are of central relevance for the management of patients with suspected COVID-19 in the ED and might imply cautious management of patients with possible COVID-19 requiring emergency care, with regard to anticoagulation. 18

In particular D-dimer, aPTT, plasminogen, fibrinogen, and low platelet count have previously been suggested as parameters for risk and therapy stratification in patients with COVID-19. However, these analyses focused mainly on the severity of the disease. 3 9 10 11 12 19 20 With regard to D-dimer, we detected only subtle increases in concentration between COVID-19-positive and -negative patients at presentation to the ED. Indeed, we detected a median value of D-dimer of 1.1 mg/L in PCR-negative patients and 1.0 mg/L in proven COVID-19 patients. Pathological values, surpassing the cut-off of 0.5 mg/L, were observed in 14 of 17 (82%) and 30 of 41 (73%) in COVID-19 and non-COVID-19 patients, respectively. This clearly indicates that D-dimer cannot be used with regard to risk stratification in a cohort of patients with suspected COVID-19 at admission. Nonetheless, exceptionally elevated D-dimer in patients with COVID-19 may result from plasmin-associated hyperactive fibrinolysis, pointing toward an interaction between D-dimer and the plasminogen/plasmin system. 21 Indeed, on a molecular level, plasmin cleaves the SARS-CoV-2 spike protein thereby leading to enhanced infectivity and virulence. Thus, plasminogen, the fibrinolytic zymogen which is proteolytically activated by T-PA to plasmin, was suggested as biomarker and therapeutic target in COVID-19 patients. While reduction in plasminogen levels in the blood may represent a biomarker for disease severity, improved oxygenation was demonstrated after plasminogen inhalation in a case series of 13 COVID-19 patients. 22 23 Indeed, in our cohort, 47% of patients with COVID-19, and 20% of patients without COVID-19 displayed elevated plasminogen levels. Given enhanced plasminogen as potential susceptibility factor for COVID-19, we detected increased levels in *n* = 8/17 versus *n* = 8/41 and decreased levels in *n* = 3/17 versus 7/41 in COVID-19 versus non-COVID-19. However, as for D-dimer, the large overlap of pathological and physiological levels within the two study groups allows only incremental usefulness in clinical practice. Numerous studies aimed at establishing T-PA, which activates plasminogen to plasmin, as well as PAI-1, which acts as an upstream inhibitor of T-PA, as potential pathognomonic analytes with regard to COVID-19 disease severity. Indeed, plasma levels of T-PA were found to be sixfold elevated in SARS-CoV-1 infection, suggesting potential endothelial injury. 24 A study in ICU COVID-19 patients demonstrated that non-ICU patients were characterized by both lower T-PA and PAI-1 levels than ICU patients. 25 In contrast, two case series with a total of eight cases of COVID-19 patients with ARDS showed beneficial results due to T-PA administration. 26 27 In our cohorts in the ED, we found differences in the proportion of patients with pathological elevations between the four study groups for both T-PA and vWF. However, while 24% ( *n* = 4/17) of COVID-19 and only 5% ( *n* = 2/41) of non-COVID-19 patients displayed elevated T-PA, this difference did not quite reach statistical significance ( *p* = 0.055). Interestingly, all COVID-19 patients with elevated T-PA were among the COVID-19 ICU subgroup ( *p* = 0.01), implying applicability as a biomarker for severity. In contrast, PAI-1 did not differ amongst our groups at admission. Strikingly, all patients with COVID-19, displayed vWF-antigen levels above the cut-off, compatible with an acute-phase reaction. However, also in non-COVID-19 patients 100% of ICU-admitted and 77% in non-ICU patients were characterized by elevated vWF.

Shorter aPTT was shown in patients with severe courses in a pooled meta-analysis comprising seven single- and multicenter studies (95% confidence interval [CI] −1.94 to 0.33). 19 Of note, while some studies reported a reduced aPTT in severe cases (even within these studies numerous cohorts did not report on reduced aPTT levels), 28 some reports showed the opposite, 29 pointing toward large heterogeneity with regard to aPTT. Further, also PT was prolonged in a meta-analysis comprising of 10 studies. However, again, some studies did not report on disturbed PT values. 28 In our cohort, focusing on coagulation analysis at admission, we detected neither altered INR nor aPTT when comparing patients presenting at the ED with suspected COVID-19 and proven COVID-19, thus in patients depicting comparable symptoms.

In contrast to aPTT, fibrinogen levels showed a rather congruent picture when viewed as risk stratification between mild and severe course. Thus, most studies point toward higher fibrinogen levels in severely affected COVID-19 patients, 30 with other studies not reporting on significant differences. 3 Elevations of fibrinogen might occur as a result of increased inflammatory activity, as fibrinogen seems to correlate with IL-6 levels at admission. 31 This demonstrates potential relevance of fibrinogen as a marker of risk stratification of COVID-19 at early stages of the disease, however, this consideration ignores the fact that a large number of patients presenting to the ED may have elevated fibrinogen levels, especially if they are characterized by respiratory symptoms and thus by an acute phase reaction. As a matter of fact, in our cohort, no statistically significant differences were detected between the two study groups, neither in median absolute values (5.2 g/L in COVID-19, 5.0 g/L in non-COVID-19, *p* = 0.82), nor in proportion of patients with elevated levels (71% in COVID-19, 71% in non-COVID-19, *p* = 1.00).

Lower platelet counts have been suggested as a biomarker in critically ill COVID-19 patients, 32 although it was highlighted that the time point of blood analysis seems crucial with relatively normal values in the early phase of the disease. 33 Here we report no differences in platelet counts at admission between COVID-19 and non-COVID-19 patients, with median 199.5 and 236.0, respectively ( *p* = 0.16). This accounted also for differences between all subgroups.

Others have shown a high percentage (91%) of positivity for lupus anticoagulant in COVID-19 patients that were also characterized by prolonged aPTT. 15 We detected in our cohort of patients in the ED rather moderate frequencies of positive lupus anticoagulant with no significant difference between the COVID-19 compared with the non-COVID-19 patient group, which might reflect different technical analytical processes. Nonetheless, the observed prevalence of lupus positivity in our cohort is in agreement with recent other findings, showing moderate frequencies of positive lupus anticoagulant of 22.2% in COVID-19 patients. 34

Finally, other laboratory findings besides coagulation parameters may hint toward different pathologies in COVID-19 compared with non-COVID-19 patients. Thus, we found significantly elevated lipase as early as at admission in COVID-19 with 38 versus 6% ( *p* = 0.01) of patients with values above the cut-off of 60 U/L. Lipase elevations in COVID-19 patients have been recognized earlier, with the pathophysiological basis as well as the clinical relevance yet to be determined. 35 Lactate showed no difference between COVID-19 and non-COVID-19 patients, but within the two COVID-19 subgroups, lactate was significantly higher in patients that were subsequently admitted to the ICU, reflecting potential septic shock, and applicability for risk stratification. In addition, lactate may be beneficial for differentiation, among severely ill patients with potential COVID-19, with higher levels among COVID-19 ICU patients than non-COVID-19 ICU patients ( *p* = 0.06).

To our knowledge, this is the first thorough description of combined routine and nonroutine coagulation analyses comparing patients suspected for COVID-19 and proven SARS-CoV-2 infected patients at admission to the ED. In light of the recent attempts to outline optimal anticoagulation regimes in COVID-19, 20 36 37 our findings underline the difficulty to identify patients who will benefit from anticoagulation at an early stage. Our data might indicate that continuous monitoring of coagulation seems mandatory in patients with proven COVID-19 infection or in those patients where infection cannot yet be reliably ruled out. It remains a challenging task to identify those patients in need for anticoagulation and those where anticoagulation is rather reluctantly indicated, as coagulation is not yet clearly disturbed in the early stages of the disease, i.e., at the time point of admission. Thus, the indication for administration of anticoagulants must carefully weigh up the general risk of anticoagulation in this cohort. 38

Taken together, our analyses point toward only subtle changes in coagulation early in the disease progression, and, in particular, no major differences between patients diagnosed with COVID-19 compared with those patients with comparable symptoms but negative for SARS-CoV-2 at presentation to the ED. This highlights moreover that coagulation parameters should be closely monitored during disease progression, since no early indications of disturbed coagulation are detectable.
