# Supplementary material for: A Matter of Caution: Coagulation Parameters in COVID-19 Do Not Differ from Patients with Ruled-Out SARS-CoV-2 Infection in the Emergency Department
Source: TH Open. 2021 Feb 6;5(1):e43–55. doi: 10.1055/s-0040-1722612 (PMC7867413; doi:10.1055/s-0040-1722612)
Supplement: Supplementary file 1 — Supplementary Material [file 10-1055-s-0040-1722612-s200100.pdf]

Supplementary Table S1 List of abbreviations

Abbreviation	Full name
CRP	C-reactive protein
PCT	procalcitonin
eGFR	estimated glomerular filtration rate
UREA	blood urea
LDH	lactate dehydrogenase
CK	creatinine kinase
AST	aspartate transaminase
ALT	alanine transaminase
GGT	gamma-glutamyl transferase
AP	alkaline phosphatase
LIP	lipase
TBIL	total bilirubin
TSHB	thyroid stimulating hormone in blood
WBC	white blood cells
RBC	red blood cells
Hb	hemoglobin
MCV	mean corpuscular volume
MCH	mean corpuscular hemoglobin
MCHC	mean corpuscular haemoglobin concentration
RDW	RBC distribution width
PLT	platelets
MPV	mean platelet volume
DDIM	D-dimers
INR	international normalized ratio
aPTT	activated partial thromboplastin time
TT	thrombin time
AT	antithrombin
F5	factor 5
FIB	fibrinogen
PLG	plasminogen
A2AP	alpha-2 antiplasmin
PROTC	protein C
PROTS	protein S
APCR	activated protein C resistance
VWF	von Willebrand factor
RICOF	Ristocetin cofactor
T-PA	tissue plasminogen activator
PAI-1	plasminogen activator inhibitor-1

Supplementary Table S2 List of laboratory parameter reference ranges

	Male	Female	Units
CRP	<5	<5	mg/L
PCT	<0.5	<0.5	µg/L
LDH	135–250	135–250	U/L
CK	<190	<167	U/L
AST	<50	<35	U/L
ALT	<41	<31	U/L
GGT	8.0–61	5.0–36	U/L
AP	40–130	35–105	U/L
LIPASE	11–60	11–60	U/L
TBIL	<1.2	<1.2	mg/dL
Lactate	<20	<20	mg/dL
DDIM	<0.5	<0.5	mg/L
INR	0.9–1.25	0.9–1.25	–
APTT	26–40	26–40	seconds
AT	80–120	80–120	%
F5	60–150	60–150	%
PLG	80–120	80–120	%
A2AP	80–120	80–120	%
PROTC	70–130	70–130	%
PROTS	80–140	55–125	%
APCR	>2.9	>2.9	%
VWF-Ag	50–160	50–160	%
RICOF	50–160	50–160	%
T-PA	2–8	2–8	µg/L
PAI-1	7–43	7–43	ng/mL

 © 2021. American Academy of Audiology.
